# An Adult Female Presenting With "Scrofula-Tubercular Lymphadenitis" a Rare Encounter: A Case Report

**DOI:** 10.7759/cureus.25650

**Published:** 2022-06-04

**Authors:** Anuradha Sakhuja, Dhan B Shrestha, Ayusha Poudel, Wasey Ali Yadullahi Mir, Tilak Joshi

**Affiliations:** 1 Department of Internal Medicine, Mount Sinai Hospital, Chicago, USA; 2 Intensive Care Unit, Nepal Korea Friendship Municipality Hospital, Kathmandu, NPL

**Keywords:** developed countries, tuberculosis, lymphadenitis, lymph nodes, fine-needle biopsy

## Abstract

Cervical lymphadenitis is the most common extra-pulmonary manifestation of tuberculosis (TB). Usually, presenting with a neck mass with minimal systemic symptoms is a diagnostic challenge for physicians. Diagnosis is made by combining clinical features, microscopic and radiological imaging, and fine-needle aspiration biopsy. A biopsy is the simplest and most cost-effective means of diagnosis. We are reporting a case of a female presenting with a neck mass without systemic symptoms who were found to have lymph node TB along with active lung disease. She was treated with a nine-month course of the direct observation treatment regimen.

## Introduction

Although rare in developed nations, tuberculosis (TB) is a significant cause of morbidity and mortality in developing countries, with almost 90% of cervical lymphadenitis caused by Mycobacterium tuberculosis [1]. Tubercular lymphadenitis is the most common extra-pulmonary manifestation of TB [2]. Patients with cervical TB usually present with a neck swelling and minimal systemic symptoms like fever, night sweats, weight loss, and fatigue [3]. Since it is uncommon in developed nations, it may present as a diagnostic challenge leading to delay in diagnosis and treatment [2]. Fine Needle Aspiration Biopsy (FNAB) is the simplest and the most cost-effective modality for diagnosing this condition [3]. Even after diagnosis, the response to treatment may vary with slow or paradoxical responses in HIV-negative patients for which steroids have been recommended [4].

## Case presentation

**Figure 1:**
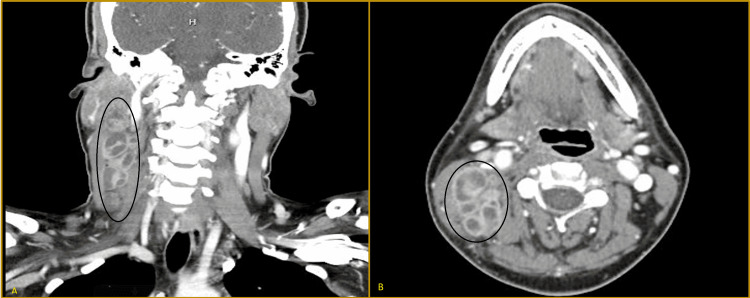
CT scan of the neck showing hypoattenuating/centrally necrotic glomerate lymph node mass (4.2 x 7.4 cm); Black circle in coronal section (A), and transverse section (B)

**Figure 2:**
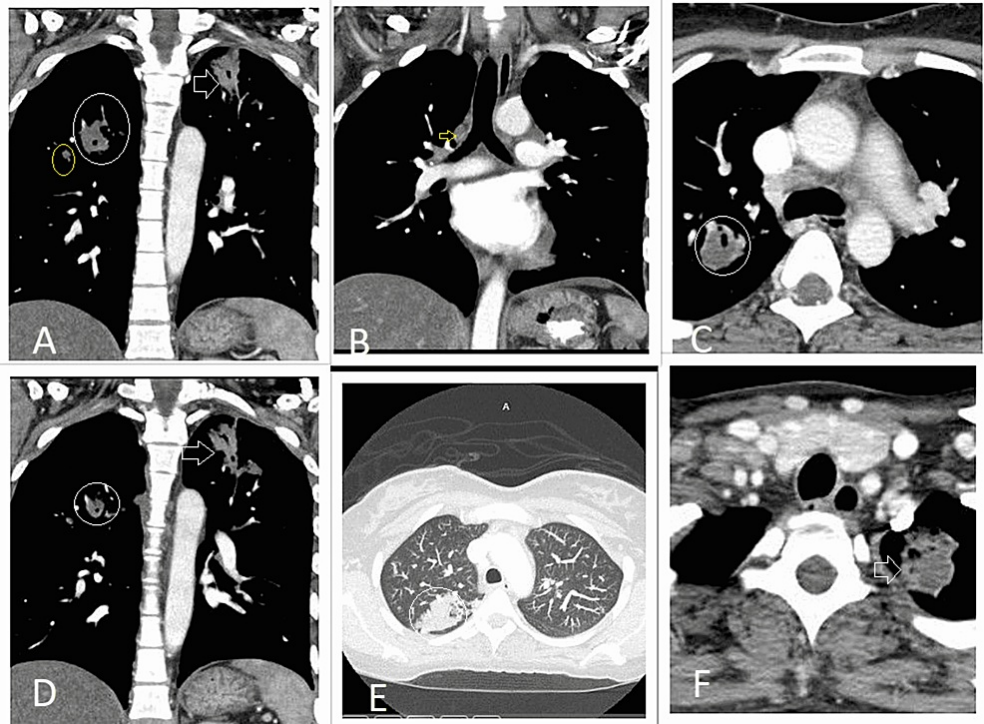
CT scan of thorax showing 22 mm focus of mass-like consolidation in the apex of the right upper lobe (white circle in A, C, D, E) with satellite lesion immediately adjacent to mass (yellow circle in A), 27 mm left apical mass like infiltrating (white arrow in A, D, F) with associated 16 mm satellite lesion and 12 mm left upper lobe lingular segmental infiltrate with pretracheal adenopathy (yellow arrow in B)

A 32-year-old African-American female with a past medical history of gestational hypertension presented with a small right side neck mass associated with neck and throat pain for two months. She had a tender, nonmobile neck swelling. A computed tomogram (CT) scan of the neck with contrast was performed, which showed lymphadenopathy in the neck with cystic versus necrotic components (Figure 1), left apical lung nodule, and right upper lobe opacity (Figure 2).

On further questioning, the patient denied any incarcerations or international travel; however, she reported a remote history of TB exposure in childhood which was treated for only one week, never completing the therapy. Her QuantiFERON test was positive. Histoplasmosis and blastomycosis tests were negative. Acid-fast bacillus (AFB) sputum cultures were ordered; however, the patient left against medical advice, denying further care. She presented a few months later again with increasing size of right neck mass and severe pain. The patient mentioned that the pain got worse with neck movement and pressure when lying down. Right neck mass was almost 5 cm, nonmobile, tender, rubber-like consistency. She was afebrile and denied night sweats, weight loss, chills, anorexia, dysphagia, odynophagia, or hoarseness. Contrast-enhanced computerized tomography (CECT) neck was repeated, and it demonstrated enlarging neck mass with necrotizing lymphadenitis. In addition, the patient had developed multiple hypoattenuating cervical lymph nodes. It also showed the presence of cavitary lesions in the left upper lobe and cavitary nodular infiltrates in the right upper lobe region. AFB sputum cultures were positive for Mycobacterium Tuberculosis complex (MTB) in two out of three samples (rifampin resistance was not detected). She underwent a fine-needle aspiration biopsy of the right neck mass which was negative for malignancy; however, it was positive for granulomatous inflammation, not enough sample for MTB. The patient was started on anti-TB therapy for an entire course of nine months under a directly observed treatment short-course regimen for active lung and lymph node TB. The patient completed nine months, and at the most recent outpatient clinic follow-up, her right neck mass has resolved.

## Discussion

The most common cause of peripheral lymphadenitis due to tuberculosis is the reactivation of latent infection [5]. Scrofula may develop due to draining into the cervical lymph nodes from the primary site of infection. In children, the infection can directly spread from the oropharyngeal mucosa to the cervical lymph nodes [6].

The most common presenting symptom in adults is chronic lymphadenopathy without systemic symptoms [7]. On inspection, it appears like a swollen lymph node, oval or spherical, with or without draining sinuses [5]. In our case, the lymph nodes seemed to be supple and swollen without draining sinuses. In addition, compressive symptoms like dysphagia and hoarseness can sometimes be present but were absent in our patient.

To make a diagnosis of tubercular lymphadenitis, a thorough history and physical examination coupled with staining and culture for AFB, radiological imaging, and fine-needle aspiration have to be performed [8]. Diagnosis made only based on clinical features has poor sensitivity [9]. Therefore, FNA cytology has been suggested as the screening tool for diagnosis as it has 88% sensitivity and 96% specificity, respectively [8]. Microscopic examination shows the presence of granulomatous inflammation with or without caesation in association with reactive hyperplasia, similar to our case [10]. Therefore, the FNA cytology was performed in our case to obtain a tissue biopsy that showed granulomatous inflammation with no findings suggestive of malignancy. The most common finding on CT scans is multiple low attenuation lymph nodes with peripherally enhancing thick rims. In some, it may coalesce into a necrotic mass obliterating the fascial planes [11].

A meta-analysis showed that the optimum duration for treatment of tubercular lymphadenitis is six months [12]. In the study, it was found that the relapse rate for cases treated for six months duration was 3.3% (95% confidence interval: 1.7-5.5), with a mean follow-up of 31 months after completion of treatment completion whereas those treated for nine months resulted in a relapse rate of 2.7% (95% confidence interval: 0.6-7.8), with a mean follow-up of 20 months. Our patient was started on RIPE therapy for an entire course of nine months under a direct observation treatment regimen for active lung and Lymph node TB.

## Conclusions

Scrofula is one of the most common extrapulmonary manifestations of tuberculosis. The most common symptom is chronic isolated swelling in the cervical region without systemic symptoms. FNAC is a useful screening tool for the diagnosis of TB.
